# Spatial clustering of physical activity and obesity in relation to built environment factors among older women in three U.S. states

**DOI:** 10.1186/1471-2458-14-1322

**Published:** 2014-12-24

**Authors:** Kosuke Tamura, Robin C Puett, Jaime E Hart, Heather A Starnes, Francine Laden, Philip J Troped

**Affiliations:** Department of Health and Kinesiology, Purdue University, Lambert Fieldhouse, Room 106, 800 West Stadium Avenue, West Lafayette, IN 47907-2046 USA; Maryland Institute of Applied Environmental Health, School of Public Heath, University of Maryland, College Park, MD USA; Channing Division of Network Medicine, Department of Medicine, Brigham and Women’s Hospital and Harvard Medical School, Boston, MA USA; Department of Environmental Health, Harvard School of Public Health, Boston, MA USA; Department of Kinesiology, California Polytechnic State University, San Luis Obispo, CA USA; Department of Epidemiology, Harvard School of Public Health, Boston, MA USA; Department of Exercise and Health Sciences, University of Massachusetts Boston, Boston, MA USA

## Abstract

**Background:**

Identifying spatial clusters of chronic diseases has been conducted over the past several decades. More recently these approaches have been applied to physical activity and obesity. However, few studies have investigated built environment characteristics in relation to these spatial clusters. This study’s aims were to detect spatial clusters of physical activity and obesity, examine whether the geographic distribution of covariates affects clusters, and compare built environment characteristics inside and outside clusters.

**Methods:**

In 2004, Nurses’ Health Study participants from California, Massachusetts, and Pennsylvania completed survey items on physical activity (N = 22,599) and weight-status (N = 19,448). The spatial scan statistic was utilized to detect spatial clustering of higher and lower likelihood of obesity and meeting physical activity recommendations via walking. Clustering analyses and tests that adjusted for socio-demographic and health-related variables were conducted. Neighborhood built environment characteristics for participants inside and outside spatial clusters were compared.

**Results:**

Seven clusters of physical activity were identified in California and Massachusetts. Two clusters of obesity were identified in Pennsylvania. Overall, adjusting for socio-demographic and health-related covariates had little effect on the size or location of clusters in the three states with a few exceptions. For instance, adjusting for husband’s education fully accounted for physical activity clusters in California. In California and Massachusetts, population density, intersection density, and diversity and density of facilities in two higher physical activity clusters were significantly greater than in neighborhoods outside of clusters. In contrast, in two other higher physical activity clusters in California and Massachusetts, population density, diversity of facilities, and density of facilities were significantly lower than in areas outside of clusters. In Pennsylvania, population density, intersection density, diversity of facilities, and certain types of facility density inside obesity clusters were significantly lower compared to areas outside the clusters.

**Conclusions:**

Spatial clustering techniques can identify high and low risk areas for physical activity and obesity. Although covariates significantly differed inside and outside the clusters, patterns of differences were mostly inconsistent. The findings from these spatial analyses could eventually facilitate the design and implementation of more resource-efficient, geographically targeted interventions for both physical activity and obesity.

## Background

High rates of physical inactivity and the obesity epidemic continue to pose major public health burdens that not only influence children and adults, but also affect older adults in developed countries such as the United States [1–3]. Despite the health benefits of physical activity [1], U.S. national data collected objectively with accelerometers showed that older adults attained the lowest levels of physical activity among all age groups [2]. Furthermore, a U.S. national survey from 1999–2008 on the prevalence of obesity among adults indicated that 37% of men (≥60 years; highest among all age groups) and 34% of women (≥60 years) were obese [4]. Among older adults, weight gain is associated with declines in functional performance and daily abilities, which in turn can lead to more sedentary lifestyles [5].

To address these issues, the U.S. Department of Health and Human Services [1] and the World Health Organization [6] have strongly emphasized the importance of physical activity-friendly environments [7] and neighborhoods with better access to healthy foods [8]. The influence of environmental exposures on individual health may increase with age as older adults spend longer periods of time in or near residential areas [9]. A review of the neighborhood influences among older adults indicated that neighborhood environments can affect the older population’s health and functioning [10]. The majority of the literature indicates that there are positive relationships between neighborhood built environment characteristics (e.g., land use mix, population density, street connectivity, and access to recreational facilities) and physical activity among older adults [11–14]. Certain characteristics of neighborhood environments (e.g., a higher density of fast-food restaurants) are positively associated with obesity [15, 16] and body weight [17]. In contrast, neighborhood walkability (i.e., describing the extent to which an environment is conducive to walking and an active lifestyle) and land use mix are negatively associated with obesity [13], body mass index (BMI) [18], and body weight [17] among older adults. However, results from other studies indicate null associations of neighborhood walkability, green spaces, street connectivity, and urban sprawl with BMI [19, 20] and obesity [9, 20, 21] among older adults.

The majority of the studies cited above utilized geographically referenced data (e.g., participant’s geocoded home address) in the analyses. If participants in a given study live close to each other, their corresponding environmental characteristics would tend to be more similar [22]. Thus, relationships between the built environment and physical activity and obesity are clearly embedded in a spatial context [22]. However, most built environment studies have not taken these spatial relationships into consideration in the analysis.

Spatial analytic techniques are needed to better understand the geographic patterns of physical activity and obesity in relation to the built environment. Spatial clustering analysis, which tests for unusually concentrated areas with high or low prevalence of specified outcomes, is one technique that can be used to investigate spatial patterns of physical activity and obesity. Spatial clustering techniques have been applied in studies of chronic diseases, such as certain cancers [23–29] and type II diabetes [30], in order to identify specific geographic areas where public health professionals may need to increase disease screenings and other prevention-related activities.

Recently, researchers have begun to apply spatial clustering techniques to physical activity [31–33] and weight-related outcomes, such as obesity [32, 34–36] and BMI [37, 38]. Spatial clusters were consistently identified across these studies despite differences in cluster detection methods, participant characteristics, and geographic areas [31–38]. Collectively, these studies demonstrate the utility of spatial clustering techniques for studying physical activity and obesity.

Nevertheless, these spatial clustering studies [31–38] have certain limitations. First, adjustment for the geographic distribution of covariates, sometimes referred to as spatial confounders, has been limited to age [31, 34, 37] and race [37]. Failure to examine other covariates (e.g., education and income), is a key limitation since the geographic distribution of these factors could account for spatial clusters. Additionally, only one study examined differences in participants’ built environment attributes inside and outside spatial clusters of transportation-related physical activity [31]. Lastly, investigators have not yet tested for clusters of physical activity and obesity among older adults, a population known to be at greater risk for physical inactivity [39] and obesity [40]. Therefore, the objectives of this study were to: 1) determine whether or not meeting recommended levels of physical activity and obesity were spatially clustered among older women in California, Massachusetts, and Pennsylvania; 2) examine whether the geographic distribution of demographic and health-related variables account for spatial clusters; and 3) compare demographic, health-related, and built environment attributes for participants living inside and outside spatial clusters.

## Methods

### Participants

The Nurses’ Health Study (NHS) is an ongoing cohort study that began in 1976 with 121,700 female registered nurses (ages 30–55 years at recruitment, 97% Caucasians) from 11 states. Currently NHS participants live in all U.S. states. The initial focus of the NHS study was to prospectively examine risk factors for chronic diseases, such as cardiovascular disease and cancer [41]. Participants are mailed follow-up questionnaires biennially, which assess potential risk factors and health outcomes. The current study builds on an exploratory study of NHS participants in California, Massachusetts, and Pennsylvania that involved developing objective built environment measures and testing associations with physical activity and obesity [42]. Thus, the current study involved 22,599 NHS participants from these three states who completed the 2004 NHS survey and met the following criteria: 1) had a geocoded home address; 2) had complete information on physical activity, body weight, and walking limitations; 3) reported they were able to walk; and 4) did not live in a nursing home. All procedures for this study were approved by the Institutional Review Boards at Purdue University, West Lafayette, Indiana, and the Human Subjects Committee at Brigham and Women’s Hospital, Boston, Massachusetts.

### Physical activity and obesity

Participants reported their average time per week engaged in walking for exercise or to work during the previous year. Participants were also asked to provide their walking pace (i.e., easy/casual [<2.0 mph]; normal/average [2.0-2.9 mph]; brisk [3.0-3.9 mph]; and very brisk [≥4.0 mph]). Consistent with previous NHS studies using physical activity data, walking metabolic equivalent (MET) minutes/week was calculated by multiplying duration by the assigned MET value based on reported walking pace. A binary physical activity outcome was created indicating whether the participant met the current U.S. physical activity recommendation of 500 MET minutes/week of activity via walking (i.e., equivalent to 150 minutes/week of moderate-intensity activity) [1]. Self-reported height in 1976 (last time reported by NHS participants) and weight reported in 2004 were used to calculate BMI = (weight in kg)/(height in m^2^). Obesity was defined as a BMI ≥ 30.0. Underweight (BMI < 18.5) participants were excluded from all analyses (n = 473). The reproducibility and validity of the physical activity [43] and weight [44] variables have been shown previously.

### Built environment

Eleven objective built environment variables were created using ArcGIS 9.3 software (ESRI, Redland, CA) and employed methods described more fully in earlier work [42]: population density, intersection density, diversity of facilities, and eight facility density variables. Built environment variables were created within a 1200 meter line-based road network buffer (i.e., residential buffer) that extended from the geocoded home address of each participant [42]. In the previous work by this group, they created both 800 meter and 1200 meter buffers and found that differences in built environment variables for two buffer sizes were negligible [42]. Population density was calculated as the number of persons per square kilometer of area within the buffer using Landscan data [45]. Intersection density was computed by dividing the number of 3-way or greater intersections by the total length of roads [46] within the buffer using StreetMapUSA [47]. A 2006 InfoUSA™ facility database, containing North American Industrial Classification System (NAICS) codes and longitude and latitude for each facility [48] was used to create the diversity of facilities and facility density variables within each buffer. Using five categories of facilities (food, retail, services, cultural/educational, and physical activity), diversity of facilities was calculated with an entropy formula [49, 50] that estimates the mixture of facility types. Possible scores range from 0 (no diversity) to 1 (maximum diversity). Eight facility density variables were created for retail (e.g., book store), services (e.g., post office), cultural/educational (e.g., school), physical activity (e.g., gym, golf course), as well as the density of food facilities further classified into four different types of densities, including fast-food restaurants, full-service restaurants (e.g., table-service restaurant), convenience stores, and grocery stores (e.g., supermarkets). These variables were calculated by dividing the number of facilities by kilometers of road within each 1200 meter buffer.

### Covariates

A number of socio-demographic and health-related factors were examined as potential spatial confounders. For each covariate, values were averaged for all participants in a given county, resulting in one aggregate value for the county. Individual-level socio-demographic variables included age and both nurse’s and husband’s education (only assessed in 1992). At the census tract level, socio-demographic variables included proportion of the population without a high school education and median family income. Health-related variables consisted of physical activity (yes/no: meeting or not meeting physical activity recommendations), obesity (yes/no: obese or not obese), walking limitations (yes: limited a lot or a little for walking from one to several blocks; no: not limited at all), smoking status (past, current, never), history of chronic diseases (yes/no; had heart disease, cancer, diabetes), and the Alternate Healthy Eating Index (AHEI assessed in 2002, a higher value indicating healthier eating), which estimates adherence to U.S. dietary guidelines [51]. The four continuous covariates, including age, proportion of the population without a high school education, median family income, and AHEI, were expressed as quintiles. Quintiles are defined as a five-level categorical covariate. These percentile ranges are: 0–20, 20.1-40, 40.1-60, 60.1-80, and 80.1-100.

### Statistical analyses

A spatial scan statistic [52, 53] based on the Bernoulli model was used to separately test for county-level spatial clustering of women meeting current physical activity recommendations and obesity. Unadjusted tests for clustering were conducted separately for participants in each of the three states. The null hypothesis was that no spatial clusters of physical activity and obesity would be detected [52, 53]. If the null hypothesis was rejected, this was interpreted to mean that participants inside of the cluster have a higher or lower likelihood of meeting physical activity recommendations or being obese, compared to participants outside of clusters. A relative risk (RR) was generated for each cluster along with a radius of the cluster. Calculations of the sizes and locations of the clusters were based on the centroids of each county. Tests for clustering were then conducted adjusting for the geographic distribution of one covariate at a time, including demographic and health-related covariates (i.e., test for spatial confounding). This analytic approach was used due to the challenge of interpreting clustering results when more than one covariate was included. In other words, in cases where a cluster was altered by covariate adjustment, it would not be possible to determine which covariate was affecting the cluster (e.g., its size or location). This approach is consistent with the recent clustering research on active transportation and obesity [31, 37]. Age, nurse’s and husband’s education, educational attainments and median household income at the census tract level, walking limitations, previous chronic disease and obesity were included as covariates in physical activity analyses. For obesity analyses, covariates were age, nurse’s and husband’s education, educational attainments and median household income at the census tract level, walking limitations, previous chronic diseases, AHEI, smoking status, and physical activity. Since potential effects of the neighborhood built environment on weight-status may take longer to appear than the effects on physical activity behaviors, obesity analyses were restricted to women who had lived at their address ≥ 4 years (N = 19,448). Obesity analyses with the full sample were also performed. However, the differences in locations and sizes of the clusters were minor.

Monte Carlo testing was utilized to determine statistical significance of clusters. Statistical significance of the clusters was defined as a p-value less than 0.05 [52, 53]. To better understand the characteristics of physical activity and obesity clusters, socio-demographic, health-related, and objective built environment characteristics of participants were compared inside and outside the clusters using t-tests for continuous variables and chi-square tests for categorical variables. Socio-demographics, health-related factors, and built environment attributes were compared between participants living inside and outside clusters. Analyses were conducted with SaTScan™ version 9 and SAS version 9 for UNIX. Maximum window sizes were tested from 10-50% (in 10% increments) of participants at risk. Since these different window sizes did not affect the results, all reported results were based on the 30% maximum window size.

All analyses were carried out at the county level to maximize available cases and controls. According to SaTScan guidelines [54], if cases or controls are missing in a given row of data within a county, that row of data must be deleted to properly run SaTScan. To avoid further missing data caused by using finer geographic scales, the county boundary was used. Missing data at a finer scale would reduce the analytic sample and might distort the development of a spatial cluster due to artifacts of the missing data [54].

## Results

### Participant characteristics

The average age of participants in 2004 was 69.9 ± 6.8 years and was similar for women living in Massachusetts, Pennsylvania, and California. Overall, 23% of the women met current physical activity recommendations via walking (25.6% in California, 24.0% in Massachusetts, and 20.2% in Pennsylvania). Approximately 21% of participants were obese (16.8% in California, 21.8% in Massachusetts, and 24.4% in Pennsylvania).

### Spatial clusters of physical activity

**Figure 1:**
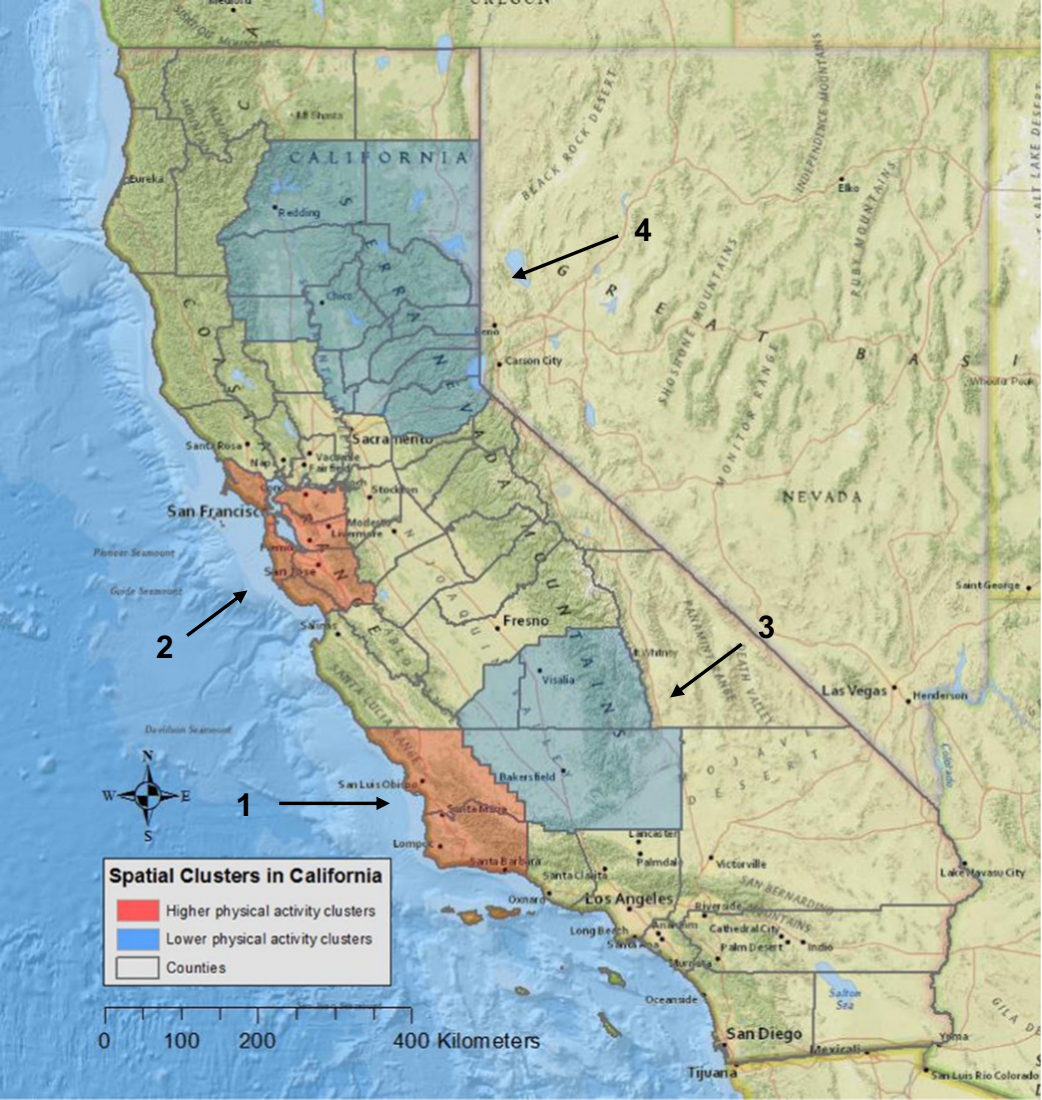
**Spatial clusters of higher and lower likelihood of women meeting physical activity recommendations in California.** The red color represents higher physical activity levels (clusters 1 and 2), whereas blue represents lower physical activity levels (clusters 3 and 4). All clusters are from unadjusted tests. Since the analyses were conducted at the county-level, clusters were visualized using a county boundary. The radius for each cluster was reported in Table 1.

Spatial clusters of women meeting physical activity recommendations via walking were identified in California and Massachusetts, but not in Pennsylvania. In California, four statistically significant spatial clusters of physical activity were identified (Table 1 and Figure 1).

**Table 1:** **Characteristics of spatial clusters of physical activity in California and Massachusetts and obesity in Pennsylvania**

	Area: Counties	Radius (km)	Participants	Cases ^a^	Relative risk	P-value
**Physical activity clusters in California**						
Cluster 1	**Coastal area**: San Luis Obispo, Santa Barbara	96.74	232	88	1.51	0.0024
Cluster 2	**Bay Area**: San Francisco, Santa Clara, Santa Cruz, Alameda, San Mateo, Marin, Contra Costa	73.19	1837	527	1.17	0.035
Cluster 3	**South inland**: Tulare, Kern Kings	121.09	129	14	0.42	0.0027
Cluster 4	**North inland**: Lassen, Shasta, Tehama, Plumas, Butte, Glenn, Sierra, Yuba, Nevada, Placer, Sutter, El Dorado	139.21	385	71	0.71	0.047
**Physical activity clusters in Massachusetts**						
Cluster 5	**Cape Cod**: Barnstable, Dukes, Nantucket	50.67	427	138	1.39	0.0003
Cluster 6	**Boston**: Suffolk	0^b^	122	43	1.48	0.053
Cluster 7	**Central/Western Massachusetts**: Berkshire, Franklin, Hampshire, Hampden Worcester	117.08	1432	306	0.86	0.06
**Obesity clusters in Pennsylvania**						
Cluster 8	**Western Pennsylvania**: Allegheny, Armstrong, Beaver, Butler, Cambria, Clarion, Forest, Indiana, Jefferson, Lawrence, Venango, Washington, Westmoreland	82.93	2424	657	1.17	0.029
Cluster 9	**Near Philadelphia**: Montgomery, Chester, Delaware	36.54	1335	268	0.8	0.01

^a^Cases are defined as participants meeting physical activity recommendations and as obese participants.

^b^Since Suffolk County was the only county identified as cluster 5, the radius was 0.

Participants inside clusters 1 and 2 had a 51% (RR = 1.51, p = 0.0024) and 17% (RR = 1.17, p = 0.035) higher likelihood of meeting physical activity recommendations, respectively, as compared to participants outside of clusters. In contrast, participants inside clusters 3 and 4 had a 58% (RR = 0.42, p = 0.0027) and 29% (RR = 0.71, p = 0.047) lower likelihood of meeting recommendations, respectively, relative to women living outside of clusters. Separately, participant’s and husband’s education, and obesity fully accounted for both clusters 2 and 4. Adjusting for other covariate adjustments, the size or location of the clusters changed. For instance, when adjusting for age, husband’s education, and obesity, cluster 1 became larger and cluster 3 became smaller. When adjusting for walking limitations, cluster 2 became smaller and the location moved to somewhat north in the San Francisco Bay Area. Adjusting for previous chronic diseases had little effect on the size or location of the clusters 1–3 in California.

**Figure 2:**
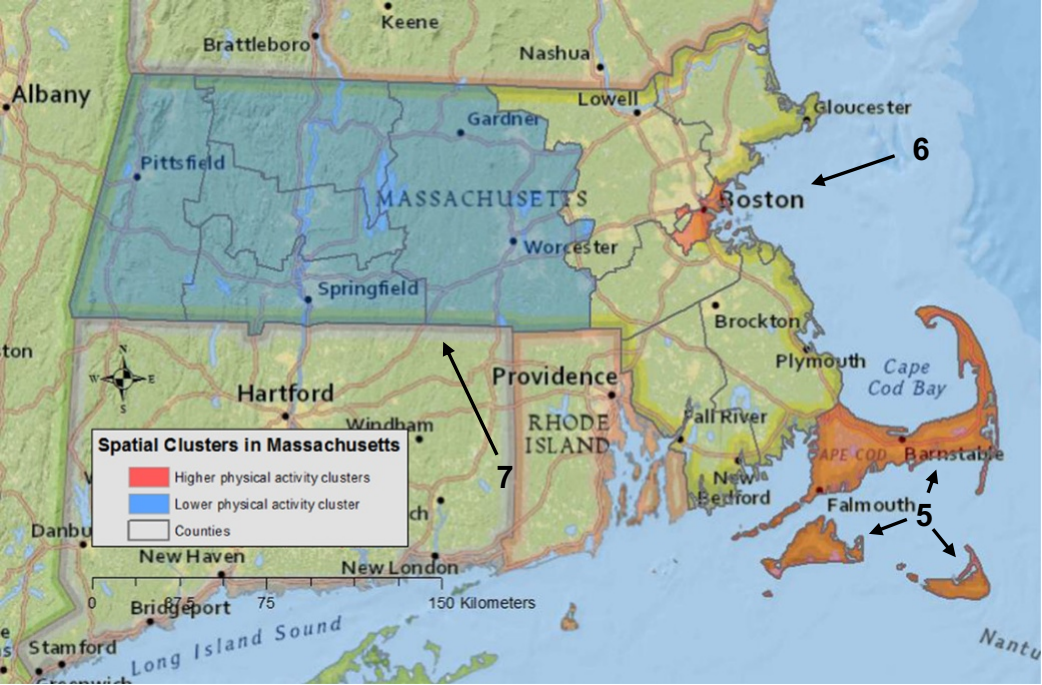
**Spatial clusters of higher and lower likelihood of women meeting physical activity recommendations in Massachusetts.** The red color represents higher physical activity levels (clusters 5 and 6), whereas blue indicates a lower physical activity level (cluster 7). All clusters were from unadjusted tests. Since the analyses were conducted at the county-level, clusters were visualized using a county boundary. The radius for each cluster was reported in Table 1.

In Massachusetts, one statistically significant cluster of physical activity and two borderline statistically significant clusters were detected (Table 1 and Figure 2). Participants inside clusters 5 and 6 had 39% (RR = 1.39, p = 0.0003) and 48% (RR = 1.48, p = 0.053) higher likelihood of meeting recommendations, respectively, compared to women outside of clusters. Participants inside cluster 7 had a 14% (RR = 0.86, p = 0.060) lower likelihood of meeting physical activity recommendations compared to participants outside the cluster. Adjusting for covariates had no effect on the three spatial clusters of physical activity in Massachusetts.

### Spatial clusters of obesity

**Figure 3:**
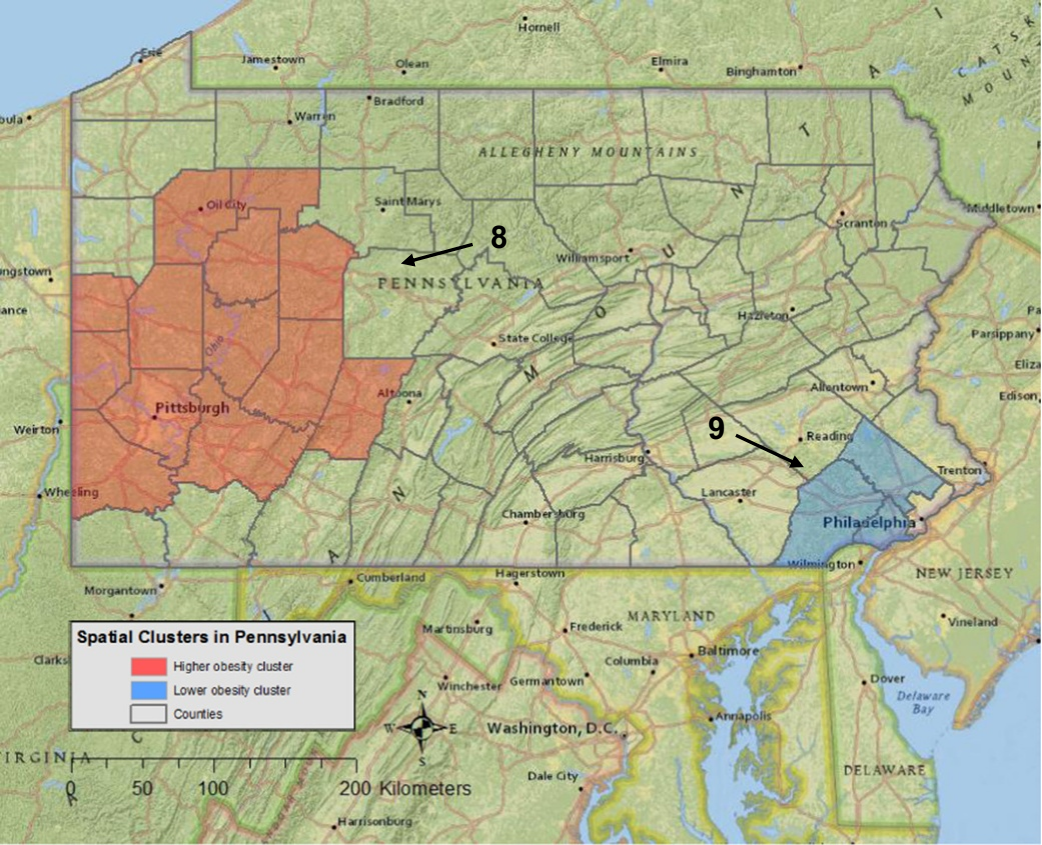
**Spatial clusters of higher and lower likelihood of obesity in Pennsylvania.** The red color represents a higher obesity level (cluster 8), whereas blue indicates a lower obesity level (cluster 9). Both clusters are from unadjusted tests. Since the analyses were conducted at the county-level, clusters were visualized using a county boundary. The radius for each cluster was reported in Table 1.

Two statistically significant spatial clusters of obesity were identified in Pennsylvania (Table 1 and Figure 3), whereas no obesity clusters were identified in Massachusetts and California. Participants inside cluster 8 had a 17% (RR = 1.17, p = 0.029) higher likelihood of obesity and in cluster 9, a 20% (RR = 0.80, p = 0.010) lower likelihood of obesity, as compared to participants outside of clusters. None of the covariate adjustments accounted for the two spatial clusters of obesity in Pennsylvania, nor did these adjustments affect the size or location of the two clusters, except for four cases. For instance, when adjusting for age, the proportion of the population without a high school education, median family income, and AHEI, cluster 9 became slightly smaller, but was at the same location.

### Comparison of demographic and health-related factors inside and outside clusters

In California there were several statistically significant differences in demographic and health-related factors. However, the magnitude of the differences in some covariates (e.g., age) was relatively small and no consistent patterns in the covariates were observed, except for median family income at the census tract level (Table 2). The two low physical activity clusters 3 and 4 in California had lower family income than did areas outside the clusters.

**Table 2:** **Participant characteristics inside and outside of recommended levels of physical activity clusters in California (N = 7153)**

Factors	Higher recommended levels of PA clusters		Lower recommended levels of PA clusters		Outside clusters
	Cluster 1	Cluster 2	Cluster 3	Cluster 4	(n = 4570)
	(n = 232)	(n = 1837)	(n = 129)	(n = 385)	
**Socio-demographics**					
*Individual level*					
Age, %^a^					
57.5 – 64.9 years	15.95	21.94**	17.05	17.92	19.67
64.9 – 69.4 years	23.71	21.45	20.16	22.6	18.84
69.4 – 73.5 years	23.28	19.22	20.16	18.96	19.89
73.5 – 78.1 years	19.4	18.56	20.16	22.6	20.18
78.1 – 85.4 years	17.67	18.84	22.48	17.92	21.42
Nurse’s education, %					
RN degree	61.64	52.42	55.81	58.44	53.26
Bachelors	22.41	29.01	26.36	27.01	26.87
Graduate degree	8.19	11.7	6.98	8.83	11.9
Missing	7.76	6.86	10.85	5.71	7.96
Husband’s education, %					
High school graduate or less	28.88	22.81**	28.68	29.09	26.37
Bachelors	27.16	24.93	18.6	28.83	24.86
Graduate degree	25.43	29.23	23.26	23.12	25.73
Missing	18.53	23.03	29.46	18.96	23.04
*Census tract level*					
Proportion of population without high school education, %^a^					
0 – 20%	4.74***	33.86***	2.33***	13.25***	16.24
20.1 – 40%	28.02	26.84	5.43	14.03	17.79
40.1 – 60%	24.57	17.15	17.83	26.23	20.46
60.1 – 80%	31.47	14.53	26.36	28.83	20.72
80.1 – 100%	11.21	7.62	48.06	17.66	24.79
Median family income, %^a^					
$18917 – 50034	18.10***	2.67***	54.26***	41.30***	24.25
$50034 – 61942	35.34	7.57	27.91	37.92	22.47
$61942 – 76251	29.31	16.82	10.85	7.01	22.21
$76251 – 94702	13.36	28.31	3.88	9.61	18.32
$94702 – 200001	3.88	44.64	3.1	4.16	12.76
**Health-related factors**					
Walking limitations, %					
Yes	25.43*	26.29***	37.21	32.73	33.13
No	74.57	73.71	62.79	67.27	66.87
Previous chronic diseases, %					
Yes	31.47	28.91***	34.11	33.77	34.42
No	68.53	71.09	65.89	66.23	65.58
Walking MET min/wk, mean (SD)	533.40 (607.40)***	431.50 (586.10)***	216.60 (339.20)***	331.60 (505.00)	374.40 (540.10)
BMI, mean (SD)	25.64 (4.27)	25.59 (4.73)*	26.60 (4.99)	26.18 (5.21)	25.89 (4.75)
**Built environment, mean (SD)**					
Population density^b^	1218.90 (812.00)***	2252.40 (1768.20)***	1358.50 (942.30)***	743.50 (748.30)***	2003.20 (1335.80)
Intersection density^c^	3.98 (1.11)*	4.41 (0.89)***	3.73 (1.21)***	3.22 (1.23)***	4.14 (1.04)
Diversity of facilities^d^	0.47 (0.34)**	0.59 (0.29)***	0.46 (0.34)**	0.28 (0.33)***	0.55 (0.31)
Facility density (total)^e^	1.31 (1.56)*	1.89 (2.25)***	0.90 (0.97)***	0.64 (1.21)***	1.59 (1.82)
Retail	0.42 (0.60)***	0.70 (0.98)***	0.29 (0.40)***	0.22 (0.49)***	0.59 (0.80)
Services	0.08 (0.15)	0.09 (0.15)***	0.04 (0.07)***	0.03 (0.10)***	0.07 (0.14)
Cultural/educational	0.31 (0.31)	0.36 (0.32)***	0.21 (0.21)***	0.14 (0.22)***	0.29 (0.27)
Physical activity	0.05 (0.09)	0.08 (0.10)***	0.04 (0.05)***	0.03 (0.06)***	0.06 (0.09)
Fast-food restaurants	2.48 (3.77)*	4.20 (7.68)***	1.43 (2.09)***	1.00 (2.58)***	3.14 (5.33)
Full-service restaurants	0.88 (1.49)	0.88 (1.47)***	0.87 (1.59)	0.41 (1.52)***	1.04 (1.66)
Convenience stores	0.21 (0.42)	0.21 (0.42)	0.28 (0.45)	0.16 (0.45)**	0.23 (0.43)
Grocery stores	0.37 (0.67)	0.41 (0.72)**	0.17 (0.37)***	0.13 (0.46)***	0.35 (0.65)

**Note:** P-values are based on the t-test for continuous variables and chi-square test for categorical variables. The values are compared between participants in a specific cluster and those outside the cluster. SD = standard deviation. PA = physical activity. *p < 0.05; **p < 0.01; ***p ≤ 0.001.

^a^A five-level categorical covariate expressed as quintiles.

^b^Population density (number of persons per km^2^ of area within residential buffer) was averaged inside and outside of clusters.

^c^Intersection density (number of intersections divided by total road length within residential buffer) was averaged inside and outside of clusters.

^d^Diversity of facilities within residential buffer (ranging from 0 [no diversity] to 1 [max diversity]) was averaged inside and outside of clusters.

^e^Facility density (number of facilities divided by kilometers of road within residential buffer) was averaged inside and outside of clusters.

In Massachusetts, there were statistically significant differences in demographic and health-related factors (Table 3). For example, educational attainments at the census tract level was significantly greater inside high physical activity cluster 5, compared to outside this cluster; and it was significantly lower in clusters 6 and 7, compared to outside these clusters. The results are inconsistent that higher education might contribute to the development of high physical activity cluster 5, but not in cluster 6. Census tract level median family income was significantly lower inside high and low physical activity clusters 5–7.

**Table 3:** **Participant characteristics inside and outside of recommended levels of physical activity clusters in Massachusetts (N = 5329)**

Factors	Higher recommended		Lower recommended	Outside clusters
	levels of PA clusters		levels of PA clusters	
	Cluster 5	Cluster 6	Cluster 7	(n = 3348)
	(n = 427)	(n = 122)	(n = 1432)	
**Socio-demographics**				
*Individual level*				
Age, %^a^				
57.5 – 62.4 years	12.88***	22.95	19.41**	20.91
62.4 – 66.4 years	17.8	19.67	17.6	21.21
66.4 – 70.7 years	21.55	20.49	19.76	20.13
70.7 – 75.7 years	23.19	18.03	20.95	19.27
75.7 – 83.4 years	24.59	18.85	22.28	18.49
Nurse’s education, %				
RN degree	65.11	56.56	71.37***	65.29
Bachelors	18.74	21.31	11.8	17.89
Graduate degree	8.43	9.02	8.45	8.99
Missing	7.73	13.11	8.38	7.83
Husband’s education, %				
High school graduate or less	25.29	22.13	38.06***	30.35
Bachelors	25.53	25.41	22	25.81
Graduate degree	23.42	24.59	17.04	20.58
Missing	25.76	27.87	22.91	23.27
*Census tract level*				
Proportion of population without high school education, %^a^				
0 – 20%	29.51***	12.30***	8.10***	24.07
20.1 – 40%	27.87	5.74	14.46	22.1
40.1 – 60%	20.61	18.03	17.81	20.58
60.1 – 80%	17.33	22.13	30.03	16.16
80.1 – 100%	4.68	41.8	29.61	17.08
Median family income, %^a^				
$17246 – 55125	47.31***	34.43***	36.59***	8.87
$55125 – 64456	39.58	21.31	27.3	14.22
$64456 – 73101	6.32	21.31	19.27	21.54
$73101 – 86110	6.79	9.84	12.02	25.96
$86110 – 191062	0	13.11	4.82	29.42
**Health-related factors**				
Walking limitations, %				
Yes	32.79	30.33	37.57***	32.5
No	67.21	69.67	62.43	67.5
Previous chronic diseases, %				
Yes	33.72	31.15	28.84	29.48
No	66.28	68.85	71.16	70.52
Walking MET minutes/wk, mean (SD)	474.90 (600.50)***	484.60 (591.60)*	338.80 (516.90)	364.90 (515.70)
BMI, mean (SD)	25.92 (4.53)**	26.62 (5.67)	26.87 (5.13)	26.63 (5.02)
**Built environment, mean (SD)**				
Population density^b^	396.70 (294.00)***	5530.70 (7422.20)***	813.60 (879.70)***	1214.90 (1271.30)
Intersection density^c^	4.14 (0.95)*	6.08 (1.13)***	3.38 (1.30)***	4.01 (1.34)
Diversity of facilities^d^	0.35 (0.35)***	0.77 (0.09)***	0.44 (0.36)***	0.52 (0.33)
Facility density (total)^e^	0.69 (1.08)***	4.21 (4.75)***	0.97 (1.14)***	1.22 (1.36)
Retail	0.21 (0.42)***	1.22 (1.22)***	0.30 (0.42)***	0.41 (0.54)
Services	0.04 (0.09)***	0.24 (0.38)***	0.06 (0.11)***	0.08 (0.12)
Cultural/educational	0.13 (0.18)***	0.91 (1.05)***	0.25 (0.28)*	0.27 (0.28)
Physical activity	0.04 (0.07)***	0.12 (0.14)***	0.04 (0.07)***	0.06 (0.09)
Fast-food restaurants	1.44 (3.00)***	15.69 (27.58)***	1.53 (2.68)***	2.20 (3.52)
Full-service restaurants	0.26 (0.72)***	1.70 (2.13)***	0.53 (0.96)	0.53 (1.10)
Convenience stores	0.27 (0.64)***	2.43 (2.35)***	0.44 (0.81)	0.48 (0.76)
Grocery stores	0.14 (0.38)***	0.57 (1.02)***	0.15 (0.43)***	0.21 (0.52)

**Note:** P-values are based on the t-test for continuous variables and chi-square test for categorical variables. The values are compared between participants in a specific cluster and those outside the cluster. SD = standard deviation. PA = physical activity. *p < 0.05; **p < 0.01; ***p ≤ 0.001.

^a^A five-level categorical covariate expressed as quintiles.

^b^Population density (number of persons per km^2^ of area within residential buffer) was averaged inside and outside of clusters.

^c^Intersection density (number of intersections divided by total road length within residential buffer) was averaged inside and outside of clusters.

^d^Diversity of facilities within residential buffer (ranging from 0 [no diversity] to 1 [max diversity]) was averaged inside and outside of clusters.

^e^Facility density (number of facilities divided by kilometers of road within residential buffer) was averaged inside and outside of clusters.

In Pennsylvania, there were statistically significant higher percentages of participants in high obesity cluster 8 with walking limitations and chronic diseases, a higher percentage of participants who never smoked, as well as lower family income, compared to areas outside of clusters (Table 4). Both individual and census tract educational levels and AHEI were significantly higher in the lower obesity cluster 9 compared to outside the cluster.

**Table 4:** **Participant characteristics inside and outside of obesity clusters in Pennsylvania (N = 8598)**

Factors	Higher obesity cluster	Lower obesity cluster	Outside clusters
	Cluster 8 (n = 2424)	Cluster 9 (n = 1335)	(n = 4839)
**Socio-demographics**			
*Individual-level*			
Age, %^a^			
57.5 – 62.4 years	19.93	21.42	19.05
62.4 – 66.8 years	19.60	19.40	20.40
66.8 – 71.1 years	21.16	18.50	19.98
71.1 – 76.2 years	20.13	19.78	20.40
76.2 – 83.5 years	19.18	20.90	20.17
Nurse’s education, %			
RN degree	69.6	66.37***	72
Bachelors	13.78	14.38	12.69
Graduate degree	6.64	10.19	5.95
Missing	9.98	9.06	9.36
Husband’s education, %			
High school graduate or less	41.46	30.04***	42.28
Bachelors	20.87	25.47	20.15
Graduate degree	15.35	21.57	16.28
Missing	22.32	22.92	21.29
*Census tract level*			
Proportion of population without high school education, %^a^			
0 – 20%	21.95***	49.66***	11.94
20.1 – 40%	25.95	23.07	17.23
40.1 – 60%	22.81	11.69	20.56
60.1 – 80%	18.81	8.46	22.42
80.1 – 100%	10.48	7.12	27.84
Median family income, %^a^			
$10461 – 42667	22.57**	1.12***	23.25
$42667 – 50341	25.70	2.77	21.49
$50341 – 58152	21.20	10.94	22.32
$58152 – 70096	18.65	22.25	20.79
$70096 – 200001	11.88	62.92	12.15
**Health-related factors**			
Walking limitations, %			
Yes	35.60**	30.71	32.32
No	64.4	69.29	67.68
Previous chronic diseases, %			
Yes	33.25**	31.69	29.68
No	66.75	68.31	70.32
Healthy eating index, %^a^			
22.5 – 44.5	18.89	17.30*	19.74
44.5 – 50.8	19.64	17.30	19.22
50.8 – 56.8	19.35	19.25	18.43
56.8 – 63.8	19.02	18.43	18.64
63.8 – 93.8	16.67	22.25	18.43
Missing	6.44	5.47	5.54
Smoking status, %			
Previous smoker	47.73*	44.42*	49.37
Current smoker	43.19	47.34	43.36
Never smoked	8.99	8.09	7.11
Missing	0.08	0.15	0.17
Walking MET min/wk, mean (SD)	309.40 (492.90)	300.60 (460.60)*	331.90 (513.90)
BMI, mean (SD)	27.41 (5.32)	26.47 (5.16)***	27.18 (5.28)
**Built environment, mean (SD)**			
Population density^b^	941.60 (997.40)***	1253.70 (913.20)*	1174.90 (1525.60)
Intersection density^c^	3.90 (1.54)***	3.69 (1.16)***	4.07 (1.59)
Diversity of facilities^d^	0.50 (0.34)***	0.53 (0.35)**	0.56 (0.33)
Facility density (total)^e^	0.97 (1.08)***	1.17 (1.16)	1.18 (1.24)
Retail	0.30 (0.43)***	0.39 (0.49)	0.37 (0.47)
Services	0.06 (0.10)***	0.08 (0.13)	0.08 (0.11)
Cultural/educational	0.27 (0.26)***	0.28 (0.23)***	0.32 (0.30)
Physical activity	0.04 (0.07)	0.05 (0.08)***	0.04 (0.06)
Fast-food restaurants	1.65 (2.68)***	1.92 (2.47)**	2.20 (4.65)
Full-service restaurants	0.66 (1.26)*	0.59 (1.16)***	0.73 (1.25)
Convenience stores	0.30 (0.54)***	0.32 (0.52)***	0.46 (0.64)
Grocery stores	0.16 (0.39)***	0.26 (0.55)	0.26 (0.59)

**Note:** P-values are based on the t-test for continuous variables and chi-square test for categorical variables. The values are compared between participants in a specific cluster and those outside the cluster. SD = standard deviation. *p < 0.05; **p < 0.01; ***p ≤ 0.001.

^a^A five-level categorical covariate expressed as quintiles.

^b^Population density (number of persons per km^2^ of area within residential buffer) was averaged inside and outside of clusters.

^c^Intersection density (number of intersections divided by total road length within residential buffer) was averaged inside and outside of clusters.

^d^Diversity of facilities within residential buffer (ranging from 0 [no diversity] to 1 [max diversity]) was averaged inside and outside of clusters.

^e^Facility density (number of facilities divided by kilometers of road within residential buffer) was averaged inside and outside of clusters.

### Comparison of built environment factors inside and outside clusters

#### Physical activity outcome

In California and Massachusetts, women living in two of the four higher physical activity clusters 2 and 6, respectively, had statistically significant higher population density (e.g., 2252 versus (vs.) 2003 persons/km^2^), intersection density (e.g., 6.08 vs. 4.01), and diversity of facilities (e.g., 0.77 vs. 0.52) and facility density (consistent with higher walkability), compared to outside of clusters. Alternatively, the values for these built environment characteristics were significantly lower for women in three lower physical activity clusters (clusters 3 and 4 in California and cluster 7 in Massachusetts).

Contrary to expectations, higher physical activity cluster 1 in California and cluster 5 in Massachusetts had built environment characteristics that indicated lower walkability, in comparison to the areas outside of clusters. In the California cluster 1, which encompassed San Luis Obispo and Santa Barbara counties, values for several variables, such as population density (i.e., 1219 vs. 2003 persons/km^2^), intersection density (i.e., 3.98 vs. 4.14), and diversity of facilities (i.e., 0.47 vs. 0.55) were significantly lower than outside of clusters. This pattern existed despite the fact that women in the cluster had 159 more MET minutes/week of walking than those outside the clusters (Table 2). In Massachusetts, participants in cluster 5 (Cape Cod area) had statistically significant lower values for most built environment attributes (i.e., the differences were in unexpected directions), yet women in this cluster had 110 more MET minutes/week of walking than outside the clusters (Table 3).

#### Obesity outcome

In Pennsylvania, the values for built environment characteristics inside obesity clusters tended to be lower compared to outside the clusters, regardless of whether or not it was a higher or lower obesity cluster (Table 4). In the higher obesity cluster 8, values for built environment characteristics, such as population density (i.e., 942 vs. 1,175 persons/km^2^), intersection density (i.e., 3.90 vs. 4.07), diversity of facilities (i.e., 0.50 vs. 0.56) and most facility density variables were significantly lower than outside the cluster. Among eight statistically significant differences in built environment characteristics inside and outside the lower obesity cluster, differences in three attributes were in the expected direction was lower inside the cluster compared to outside (e.g., fast-food facility density; 1.92 vs. 2.20).

## Discussion

The present study applied spatial scan statistics to identify spatial clusters of physical activity and obesity among approximately 20,000 older women in California, Massachusetts, and Pennsylvania. High and low physical activity clusters were identified in California and Massachusetts, while none were identified in Pennsylvania. High and low obesity clusters were detected only in Pennsylvania. The majority of the adjustments for demographics and health-related factors did not fully account for physical activity and obesity clusters, suggesting that other factors may be contributing to the development of these spatial clusters. Although some statistically significant differences in demographic and health-related characteristics inside and outside of clusters were found, not all patterns in differences were consistent. Furthermore, built environment characteristics inside and outside clusters of physical activity and obesity generally showed statistically significant differences. In a number of cases, higher physical activity clusters had higher values of population density and intersection density, expected to be associated with higher walkability. This finding is supported by a previous study on spatial clustering of active transportation in California [31]. However, in several other cases, built environment factors typically associated with higher neighborhood walkability were lower in high physical activity clusters, particularly along coastal areas in California and Massachusetts.

Identification of higher physical activity clusters in areas adjacent to the ocean in California and Massachusetts is generally consistent with findings from two previous U.S. studies [31, 36]. In a recent investigation of active transportation in California, researchers detected clusters of higher transportation-related walking near coastal areas around Long Beach and Santa Monica in Los Angeles County [31]. Another study, using data from the Behavioral Risk Factor Surveillance System (BRFSS) from 2000–2006, showed higher physical activity clusters in parts of the San Francisco Bay Area, northwest coastal states (Washington and Oregon), and by Lake Michigan [36]. Collectively, the results from these recent U.S. studies [31, 36], earlier studies in Australia, which indicated a positive influence of coastal areas on physical activity [55, 56], and the present study, suggest that living near large bodies of water has a positive relationship with physical activity. However, since all of this evidence is from cross-sectional studies, the direction of these effects cannot be determined. A plausible alternative explanation is that more active, outdoor-oriented, and health conscious adults, including older adults such as those in the present study, seek to live in areas closer to lakes and oceans.

The detection of higher and lower obesity clusters among participants in western and eastern Pennsylvania contrasts findings from two recent U.S. studies that used BRFSS data [36, 37]. In one study of U.S. adults, ages 22 to 74 years, researchers applied the spatial scan statistic to data from 1999 to 2003 and detected clusters of high and low BMI prevalence in southern (e.g., Louisiana) and western (e.g., California) states of the U.S., respectively [37]. However, they found no clusters of high or low BMI prevalence in Pennsylvania [37]. In another study of U.S. adults (aged ≥18 years) investigators used the local Moran’s *I* to identify clusters using BRFSS data from 2000 to 2006 [36]. They found significantly low obesity clusters in mountain regions of the U.S. (e.g., Colorado) and in some New England (e.g., Massachusetts) states as well as high obesity clusters in southern states (e.g., Texas) [36]. However, they did not detect significant clusters of obesity in Pennsylvania [36]. The present study’s findings may vary from these previous investigations due to differences in sample characteristics (e.g., older adults, women only, predominantly white), use of different spatial analytic techniques, the geographic scope of the study area (i.e., three states vs the entire U.S.), and the scale differences for the analyses (i.e., individual’s and census tract level variables at county level analyses for each state vs. county level variables for the analyses at the entire U.S.).

Although a number of socio-demographic and health-related factors were examined as spatial confounders in the current study, there was limited evidence that these covariates accounted for spatial clusters of physical activity and obesity. The issue of spatial confounding has received little attention in previous cluster analyses of physical activity and weight status. In two investigations of active transportation and BMI, only participants’ age [31, 37] and race [37] were evaluated as potential confounders. In these studies, there was mixed evidence that age was a spatial confounder. In one study adjusting for age fully accounted for a lower BMI cluster (i.e., disappearance of the cluster after adjustment), but only partially accounted for a higher BMI cluster (i.e., size of the cluster became larger, and location moved further south) [37]. However, in a study of active transportation clusters in San Diego County in California, age adjustment did not account for clusters [31]. Race fully explained spatial clusters of high and low BMI detected in the U.S. [37]. The limited investigation of spatial confounders suggests the need for testing other types of factors that might account for spatial clusters of physical activity and obesity. For example, these studies could include psychosocial variables (e.g., social support, self-efficacy, psychosocial hazards) that have been assessed in recent built environment studies [16, 57–60] as well as eating behaviors (e.g., eating habits in the past year, eating-out behavior since it is hypothesized that obesity would be influenced by an individual’s past eating behaviors or habits) [16, 57].

To the best of this group’s knowledge, this study is only the second to compare objective built environment characteristics inside and outside of spatial clusters of physical activity and the first to do so with obesity. Generally, a mixed pattern of differences in built environment characteristics was found, in some cases consistent with what would be hypothesized (e.g., higher connectivity in higher physical activity clusters) and in others contradicting these expectations. In contrast to the present study, Huang and colleagues found a consistent and expected pattern of built environment differences inside and outside clusters, for example, where inside high active transportation clusters the values of population density and intersection density index were higher than in areas outside of clusters in Los Angeles and San Diego counties in California [31]. The findings from the present study highlight the complexity of built environment and physical activity relationships, resulting in consistent and inconsistent patterns in the built environment factors.

There were consistent patterns in the built environmental attributes in the two high physical activity clusters 2 and 6 in California and Massachusetts, respectively. The majority of the built environment variables, including population density, intersection density, diversity of facilities, and most facility densities, were consistently higher compared to outside of clusters. These two clusters were located in more populous areas (San Francisco Bay Area and Boston) compared to the other two high physical activity clusters 1 and 5. In contrast, low physical activity clusters 3, 4, and 7 were located in inland California and middle to western Massachusetts, and most of the built environment values for these clusters were consistently lower than outside of clusters. Inconsistent patterns of built environment factors across the clusters were also found, for example, the average level of walking for participants in higher physical activity cluster 1 in California with lower built environment values, including population density, intersection density, diversity of facilities and some densities of facilities (i.e., hypothetically less favorable for walking) was 102 MET minutes/week higher than for women in higher physical activity cluster 2 with higher built environment values. One possible explanation for these findings is that certain unmeasured built environment characteristics, such as availability and condition of sidewalks, aesthetics, outdoor recreational facilities including trails and parks, or neighborhood safety (e.g., crime rates), may account for the differences in walking between these two clusters. Future analyses of physical activity clusters should examine a more comprehensive list of both perceived and objective built environment variables.

The present study has several limitations. The findings may not be applicable to more diverse groups of older women in the U.S., since the sample is predominantly Caucasian, moderately well-educated, and generally aware of health issues due to their background in nursing. The walking measure did not differentiate between walking for leisure and transportation. If separate measures of walking for recreation and transportation had been available, different clusters might have been detected and patterns in built environment characteristics inside and outside of spatial clusters might have been different for the two types of walking. Thus, inconsistencies in built environment characteristics might have been observed in this study. This study examined clustering at the county level and the actual spatial clustering of physical activity and obesity may not coincide with geo-political boundaries [61, 62]. Obesity estimates may be biased since self-reported height from 1976 was used to calculate BMI, resulting in misclassifying some participants as either obese or non-obese. As individual level income was not available, median family income at the census tract level was used in the analyses. Since the geographic distribution of individual level income would differ from the distribution of median family income, this scale difference may influence the existence of the physical activity and obesity clusters. A scan statistic based on the Bernoulli model restricts the type of the covariate adjustment to only categorical variables. In the present study, continuous covariates (e.g., median family income) were categorized into quintiles. Depending on arbitrary categories for these covariates, the assessment of the spatial clusters may be impacted with respect to the size or location, or disappearance of the cluster. The results from covariates expressed as binary and quartiles were compared to those of quintile covariates. However, the differences in results were minor.

## Conclusions

The present study contributes to the sparse literature on spatial clustering of physical activity and obesity among older women, including the limited assessments of spatial confounders, and comparisons of built environment characteristics inside and outside of clusters. Although spatial clusters of physical activity were detected, the majority of the spatial confounders examined did not explain the identified clusters. The patterns of the built environment values inside and outside of clusters revealed complex relationships. Higher street connectivity was consistently found in higher physical activity clusters 2 and 6, whereas inconsistent patterns even among high physical activity clusters 1 and 2 were found (i.e., a higher level of walking for cluster 1 with unsupportive built environment characteristics, compared to cluster 2). These findings were not fully consistent with existing built environment literature. The spatial clustering methods and findings have implications for future directions in public health research and practice. For example, the findings from this study and others [31, 37] suggest that further examination of factors that contribute to the development of spatial clusters of physical activity and obesity is needed. One way to address this gap would be to examine space-time clustering of physical activity and obesity, which may have the potential to shed new light on determinants, including neighborhood built environment factors. In terms of public health practice, where surveillance data on physical activity and obesity are available along with geographic identifiers, public health officials could take advantage of existing cluster detection software, such as SaTScan™ [63], to identify clusters. Results of these spatial analyses could facilitate the design and implementation of more geographically targeted, resource-efficient interventions for both physical activity and obesity.

## Abbreviations

BMI: Body mass index
GIS: Geographic information system
RR: Relative risk
AHEI: Alternate healthy eating index
MET: Metabolic equivalent
BRFSS: Behavioral risk factor surveillance system
NHS: Nurses’ health study
vs: versus.
